# Antimicrobial Peptide Screening for Designing Custom Bactericidal Hydrogels

**DOI:** 10.3390/pharmaceutics16070860

**Published:** 2024-06-27

**Authors:** Matthias Recktenwald, Muskanjot Kaur, Mohammed M. Benmassaoud, Aryanna Copling, Tulika Khanna, Michael Curry, Dennise Cortes, Gilbert Fleischer, Valerie J. Carabetta, Sebastián L. Vega

**Affiliations:** 1 Department of Biomedical Engineering, Rowan University, Glassboro, NJ 08028, USA; reckte75@rowan.edu (M.R.); mehdibenmassaoud20@gmail.com (M.M.B.); 2 Department of Biomedical Sciences, Cooper Medical School of Rowan University, Camden, NJ 08103, USA; kaurmu89@rowan.edu (M.K.); currym75@rowan.edu (M.C.); cortesd@rowan.edu (D.C.); fleischeg0@rowan.edu (G.F.); 3 Department of Translational Biomedical Sciences, Rowan University, Glassboro, NJ 08028, USA; coplin22@students.rowan.edu; 4 Department of Biological Sciences, Rowan University, Glassboro, NJ 08028, USA; khanna48@students.rowan.edu; 5 Department of Orthopedic Surgery, Cooper Medical School of Rowan University, Camden, NJ 08103, USA

**Keywords:** antimicrobial peptides, AMP screening, hydrogels, thiol-norbornene, medical device infections

## Abstract

*Staphylococcus aureus* (*S. aureus*) is an opportunistic pathogen that lives on surfaces and skin and can cause serious infections inside the body. Antimicrobial peptides (AMPs) are part of the innate immune system and can eliminate pathogens, including bacteria and viruses, and are a promising alternative to antibiotics. Although studies have reported that AMP-functionalized hydrogels can prevent bacterial adhesion and biofilm formation, AMP dosing and the combined effects of multiple AMPs are not well understood. Here, three AMPs with different antibacterial properties were synthesized and the soluble minimum inhibitory concentrations (MICs) of each AMP against methicillin-susceptible *S. aureus* (MSSA) and methicillin-resistant *S. aureus* (MRSA) were determined. Hydrogels with immobilized AMPs at their MIC (DD_13_-RIP 27.5 µM; indolicidin 43.8 µM; P10 120 µM) were effective in preventing MRSA adhesion and biofilm formation. Checkerboard AMP screens identified synergy between indolicidin (3.1 µM) and P10 (12.5 µM) based on soluble fractional inhibitory concentration indices (FICIs) against MRSA, and hydrogels formed with these AMPs at half of their synergistic concentrations (total peptide concentration, 7.8 µM) were highly efficacious in killing MRSA. Mammalian cells cultured atop these hydrogels were highly viable, demonstrating that these AMP hydrogels are biocompatible and selectively eradicate bacteria, based on soluble checkerboard-screening data.

## 1. Introduction

Prosthetic implants and indwelling medical devices are used to replace, reinforce, or support tissues and organs, and their use has increased drastically over the past 50 years [1,2]. While medical implants have significantly improved the quality of life and prolonged the lives of many patients, implants are at risk of microbial contamination. In the United States, about 2 million hospital-acquired infections (HAIs) occur yearly, and 50–70% of such infections result from indwelling medical devices [2,3,4]. Such devices can become contaminated during the surgical procedure, or from bacterial colonization of the skin or other body sites of the patient or healthcare workers [5,6]. As there are currently no specific markers of device-related infections [2], detection is typically delayed and eventual replacement of the implant is required, involving additional costly and high-risk surgeries.

Approximately 80% of all microbial infections in the human body involve the formation of multicellular communities called biofilms [2,3,7]. Bacterial biofilms formed on the surface of medical devices make the infection inherently more difficult to treat [8]. Bacteria in a biofilm secrete an extracellular polymeric substance (EPS), which encases them in a protective matrix. This matrix makes biofilms extremely difficult to eliminate and leads to decreased susceptibility to antibiotics [6,9,10]. As biofilms mature, essential nutrients become limited and toxic byproducts accumulate, causing the surface layer of bacteria to dissociate and migrate to form new biofilms, which can cause recurrent infections in patients [11,12,13,14]. The most common biofilm-related infections on implanted medical devices are caused by the Gram-positive pathogens *Staphylococcus aureus* (*S. aureus*) and *Staphylococcus epidermidis* [15]. Staphylococci are typically part of the normal flora of human skin and account for an estimated two-thirds of all medical device infections [2,16,17]. Infection with highly drug-resistant strains, such as methicillin-resistant *S. aureus* (MRSA), severely limits treatment options with standard-of-care antibiotics [18]. Given the difficulty in detecting and eradicating biofilm-based infections, the best treatment strategy might be to prevent biofilms from forming in the first place.

Antibiotics are routinely used for empiric therapy, prophylaxis, and treatment of infections, and the use of coatings to prevent biofilm formation on medical devices has been increasingly explored in recent years [14,19]. Antibiotics such as cefazolin and vancomycin were successfully tethered to surfaces and retained their antimicrobial activity [20,21,22]. For example, Boot et al. coated a titanium rod with vancomycin-laden hydrogels and found that this prevented infection when implanted in the medullary canal of rabbit tibias inoculated with *S. aureus* [22]. Despite the excitement and promise for antibiotic infused coatings, these strategies are hindered by the continuing emergence of antibiotic and multidrug-resistant infections [21]. With the rise of antibiotic resistance among bacteria, antimicrobial peptides (AMPs) represent a viable alternative to antibiotics. AMPs are short, natural peptides that are part of the innate immune system, which inhibits and kills pathogens such as viruses, bacteria, and fungi [23,24]. The anti-biofilm activity of AMPs occurs via several mechanisms, including disruption of bacterial membrane potential, cell lysis, interruption of cell signaling, and downregulation of important biofilm-formation genes [25]. For instance, the human cathelicidin LL-37 and its derivatives prevent biofilm formation by inhibiting the initial attachment of cells to a surface [26,27,28]. P10 is a synthetic LL-37 derivative that is highly bactericidal and effective at killing MRSA biofilms in a burn wound-infection model system [27,29,30]. Indolicidin is an AMP that exhibits strong anti-biofilm activity against MRSA [31], which functions by permeabilizing the cytoplasmic membrane and disrupting membrane potentials [32,33,34,35]. DD_13_-RIP is a chimeric AMP of dermaseptin (DD_13_) and RNA III inhibiting peptide (RIP), and is effective in preventing graft-associated infections with Staphylococcal species via two mechanisms [36]. RIP inhibits quorum sensing [37], a prerequisite to biofilm formation, and DD_13_ incorporates itself into bacterial membranes resulting in cell lysis [38]. Taken together, these three AMPs with anti-biofilm properties represent promising targets for designing novel AMP-laden materials.

Biomaterials which have no intrinsic antimicrobial properties can be modified to include antimicrobial molecules such as antibiotics or silver through chemical modifications or nanoparticles [39,40]. Furthermore, functionalizing materials with AMPs is a promising approach towards mitigating medical device infections [41,42,43]. AMPs have been successfully immobilized on a variety of materials, including contact lenses, titanium oxide, and silicone surfaces [44,45,46]. There are now many materials for creating antimicrobial coatings, including hydroxyapatite, ceramic, and hydrophilic polymers, such as hydrogels [47]. For instance, hyaluronic acid-derived hydrogels are promising materials for biomedical applications due to their biocompatibility and high control over physical and biochemical properties [48]. While hyaluronic acid is bacteriostatic and does not promote bacterial adhesion, it is not inherently bactericidal and must be modified to allow for the conjugation of functional molecules including AMPs [49]. One straightforward technique to tether AMPs to hydrogel matrices is by using thiol–norbornene click chemistry reactions between norbornene-modified macromers and thiol-containing peptides [50,51]. While the use of hydrogels as delivery systems for AMPs is beginning to be explored for applications such as wound dressings and localized infection prevention [52,53,54], AMP dosing is generally not tailored for bacterial pathogens it is intended to treat, and the use of multiple AMPs tethered to hydrogels has not been extensively explored.

In this study, soluble screens using DD_13_-RIP, indolicidin, and P10 AMPs against methicillin-susceptible *S. aureus* (MSSA) and MRSA were used to design customizable hydrogels with bactericidal properties tailored to the clinical isolates tested. *S. aureus* was selected as a pathogen model for this AMP screening protocol rather than other bacterial strains because it is one of the leading causes of device-associated infections and available treatments are severely limited [4]. *S. aureus* also has high morbidity and mortality rates, and its ever-growing resistance to antibiotic treatments makes it a global public health threat [55]. To identify optimal dosing, we determined the minimum inhibitory concentrations (MICs) of each soluble AMP against clinically isolated MSSA and MRSA strains. To identify potential additive and synergistic interactions between multiple AMPs, we next examined pairwise combinatorial effects between the AMPs using checkerboard arrays. Using this information, we designed thiol–norbornene hydrogels functionalized with thiolated AMPs and evaluated their efficacy in preventing MRSA biofilm formation. We found that the soluble screening data were successful in guiding the design of hydrogels with low and optimized AMP concentrations that effectively prevented biofilm formation of the bacterial isolates tested. Our soluble screens also identified a two-AMP synergistic combination against MRSA, and the two-AMP concentration was 10- to 14-fold lower than individual AMPs at their MIC, reducing the likelihood of cytotoxicity and other downstream side effects. Human mesenchymal stem cells (MSCs) cultured on RGD-functionalized AMP hydrogels were also highly viable, demonstrating that our AMP-laden hydrogels can be tailored to prevent biofilm formation from bacterial pathogens of interest while maintaining high biocompatibility.

## 2. Results and Discussion

### 2.1. AMP Screens Used to Determine MICs of Soluble AMPs against MSSA and MRSA

In this study, DD_13_-RIP, indolicidin, and P10 were chosen as candidate AMPs due to their different mechanisms of action and anti-biofilm properties [27,33,36]. MIC is a commonly used laboratory measurement to determine the in vitro activity of an antimicrobial agent against bacteria. A lower MIC typically indicates that less of the agent is required to inhibit growth of the organism, thus implying that the lower the MIC, the more potent the agent [56]. The MICs for MSSA were 13.5 µM, 18.8 µM, and 87.5 µM for DD_13_-RIP, indolicidin, and P10, respectively (Table 1). The MICs for MRSA were over 2-fold higher for DD_13_-RIP, 2.5-fold higher for indolicidin, and 1.4-fold higher for P10 in comparison to MSSA (Table 1). The MIC values for our clinical isolates were higher than those previously reported in the literature [27,33,36], which is likely due to strain differences. It is well-known that MRSA infections are more difficult to treat than MSSA infections due to increased drug resistance [57], therefore limiting treatment options for MRSA infections. Our data demonstrate that MRSA is also more tolerant of AMPs than MSSA, based upon comparison of MIC values. It is important to note that no clinical breakpoint data are available for AMPs, so susceptibility cannot be determined. A possible explanation of increased MICs in MRSA strains is that there is some level of cross-protection provided by one or more drug-resistance mechanisms, with a possible candidate being multidrug efflux pumps [58]. Our data demonstrate that drug resistance status influences the efficacy of AMPs, highlighting the need to identify optimal effective concentrations tailored to specific bacterial strains.

### 2.2. Checkerboard Arrays Identify Additive and Synergistic AMP Combinations against S. aureus Isolates

**Figure 1:**
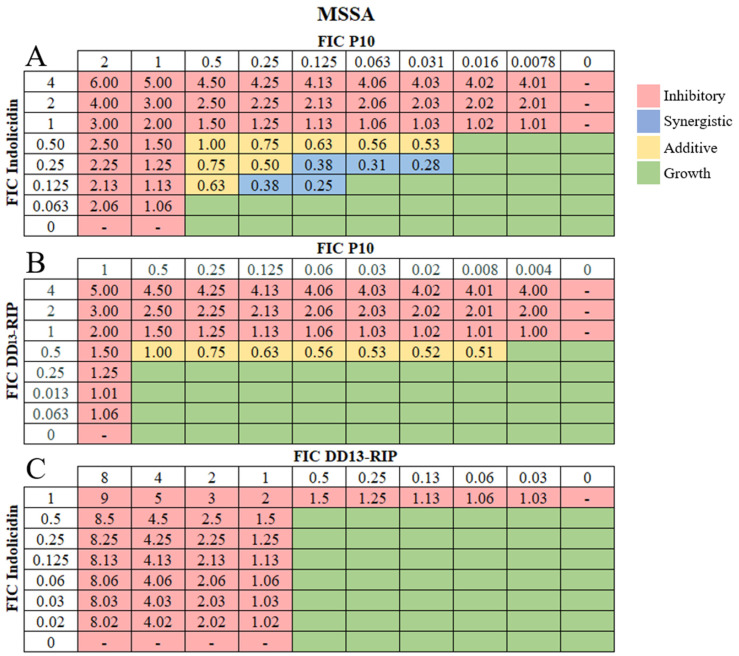
Combinatorial effects of soluble AMPs on MSSA strains. Representative checkerboard assay of fractional inhibitory concentrations (FIC) for (**A**) indolicidin and P10, (**B**) DD_13_-RIP and P10, and (**C**) indolicidin and DD_13_-RIP AMP pairs against MSSA. FICs were calculated for each drug (concentration/MIC) and added together for all wells where no growth was observed. The red-colored boxes indicate wells with no bacterial growth (OD_600_ < 0.1) and green-colored boxes indicate bacterial growth (OD_600_ > 0.1, Figure S1). The box in the bottom right corner contains no drug and serves as a growth control. Yellow-shaded boxes indicate additive interactions (FICI between 0.5–1.0) and blue boxes indicate synergistic interactions (FICI < 0.5).

**Figure 2:**
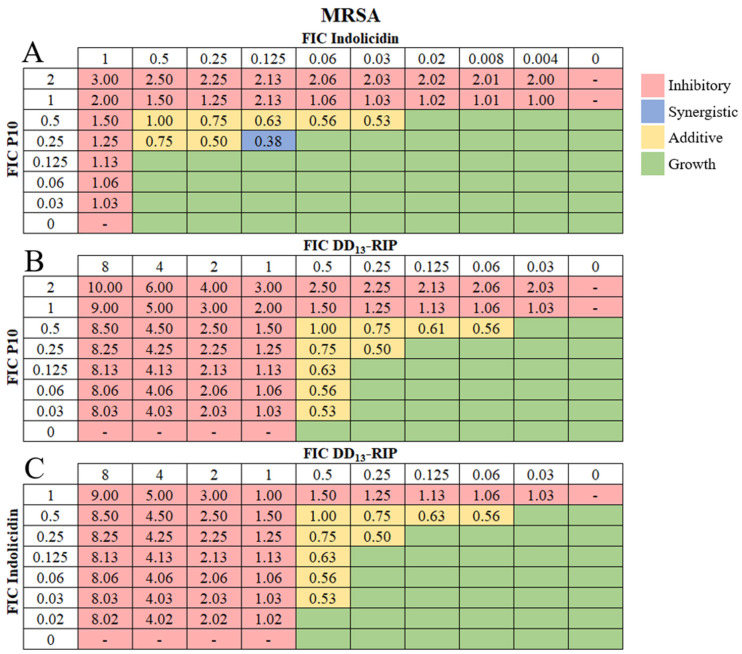
Combinatorial effects of soluble AMPs on MRSA strains. Representative checkerboard assay of fractional inhibitory concentrations (FIC) for (**A**) P10 and indolicidin, (**B**) P10 and DD_13_-RIP, and (**C**) indolicidin and DD_13_-RIP AMP pairs against MRSA. FICs were calculated for each drug (concentration/MIC) and added together for all wells where no growth was observed. The red-colored boxes indicate wells with no bacterial growth (OD_600_ < 0.1) and green-colored boxes indicate bacterial growth (OD_600_ > 0.1, Figure S2). The box in the bottom right corner contains no drug and serves as a growth control. Yellow-shaded boxes indicate additive interactions (FICI between 0.5–1.0) and blue boxes indicate synergistic interactions (FICI < 0.5).

Antibiotics used in combination allow for increased potency at lower bactericidal concentrations [59,60]. Combinatorial effects are determined in vitro using the standard checkerboard array, where two antimicrobial agents are serially diluted in different directions in a 96-well plate to assess the effectiveness of antibiotic cocktails [61]. Often, when drugs are tested together, the effect is similar to that of one of the drugs alone. An additive effect is present when the two agents have a larger effect than either alone, as if adding their efficacy together. Synergistic effects occur when their combined effect is significantly larger than either alone. Combination therapy offers an effective approach to mitigate potential cytotoxicity, adverse reactions, and the emergence of bacterial resistance to individual AMPs [62]. Thus, we used checkerboard arrays to examine pairwise combinations of DD_13_-RIP, indolicidin, and P10 AMPs for potential additive and synergistic effects against MSSA (Figure 1A–C) and MRSA strains (Figure 2A–C).

For MSSA, the combination of indolicidin and P10 yielded multiple combinatorial effects, including eight additive combinations and five synergistic combinations (Figure 1A). The concentrations of the AMPs in the synergistic combinations are significantly lower (Figure S1A) than the MIC of individual AMPs (Table 1). For DD_13_-RIP and P10, seven additive combinations were identified, but no synergistic ones (Figure 1B and Figure S1B). Surprisingly, no combinatorial effects were identified for indolicidin and DD_13_-RIP (Figure 1C and Figure S1C). For MRSA, seven additive combinations were identified between indolicidin and P10 AMPs (Figure 2A). Only one combination yielded a synergistic effect, which was at 12.5 μM P10 and 3.125 μM indolicidin (Figure S2A). We identified nine additive combinations for DD_13_-RIP in combination with either P10 or indolicidin (Figure 2B,C and Figure S2B,C). Interestingly, the combination of DD_13_-RIP and indolicidin showed additive effects against MRSA but not MSSA, demonstrating that combinatorial effects of AMPs are strain-specific. This behavior is expected, as many antibiotic combinations also show strain specificity [63], and this finding emphasizes the importance of our AMP screening approach to identify combinations tailored to specific bacterial strains. Due to clinical challenges in treating MRSA infections and our observation that more-highly soluble AMP concentrations are needed to prevent MRSA growth in vitro, we used our soluble-AMP screening data to create custom bactericidal AMP hydrogels against MRSA.

### 2.3. Single AMP-Loaded Hydrogels at the MIC Retain Antimicrobial Properties against MRSA

**Figure 3:**
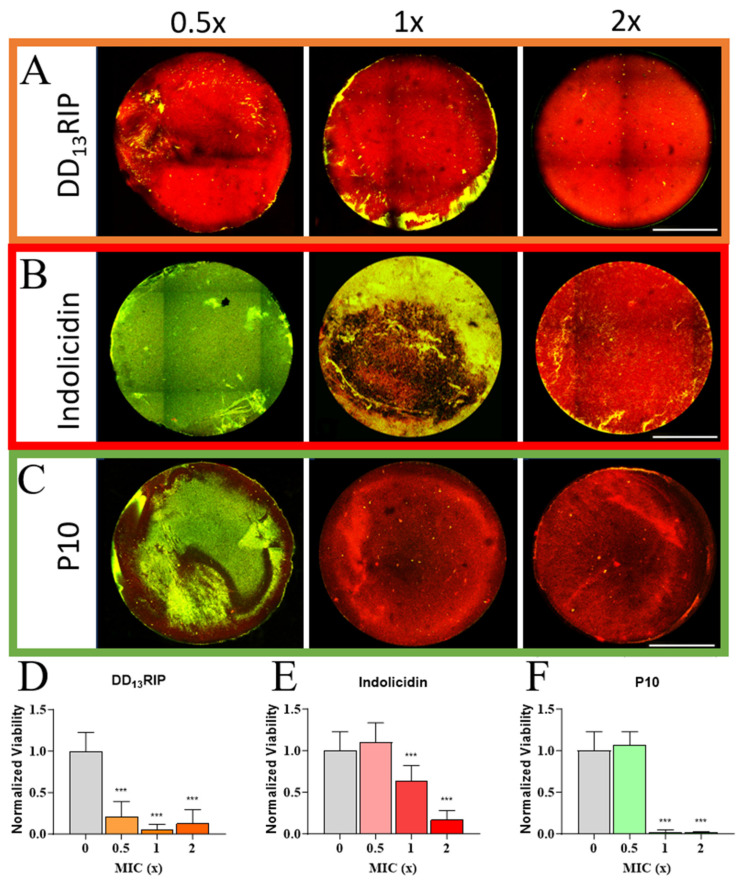
Examination of MRSA biofilm formation on different AMP-loaded hydrogels. Qualitative images of representative AMP hydrogels at 0.5×, 1×, and 2× MIC for tethered (**A**) DD_13_-RIP, (**B**) indolicidin, and (**C**) P10 AMPs. Scale bars: 1 mm. Quantitative viability analysis of MRSA on (**D**) DD_13_-RIP, (**E**) indolicidin, and (**F**) P10 AMP hydrogels. All the groups were normalized to the results of a control hydrogel group with no AMP. Significance (*** *p* < 0.001) in viability was established by comparing each test group to a control hydrogel group with no AMP (0×).

While antimicrobial peptides offer a viable replacement for antibiotics, in their soluble form, the clearance rate of fluid surrounding a wound or implant has limited their use for biomedical applications. The immobilization of AMPs to a biocompatible substrate can mitigate this dilution effect and expand the lifetime of AMPs [52]. Towards this end, we investigated the transferability of the soluble peptide concentrations determined in broth into AMP hydrogels by tethering thiolated AMPs, at concentrations equivalent to 0.5×, 1×, and 2× the MICs, to hyaluronic acid hydrogels using thiol–norbornene click chemistry reactions [50,51]. Hyaluronic acid was chosen as the hydrogel macromer due to its mammalian cell biocompatibility and bacteriostatic properties. MRSA was incubated with AMP-functionalized hydrogels (3 mm diameter, 0.5 mm thickness) for 2 days under biofilm-promoting conditions. Next, non-adherent cells were removed, hydrogels stained with a mixture of Syto-9 and propidium iodine (live-dead), and samples imaged with a confocal microscope (Figure 3A–C). A hydrogel incubated in PBS with no bacteria was also stained and imaged which showed no fluorescence, indicating that Syto-9 and propidium iodine do not stain the hydrogels (Figure S3). For DD_13_-RIP, all the AMP concentrations tested showed antimicrobial properties, evidenced by a visibly high red fluorescence, indicating dead cells (Figure 3A). The percentage of viable bacteria was calculated to be 21%, 5%, and 13% relative to AMP-free control hydrogels at 0.5×, 1× and 2× MIC, respectively (Figure 3D). The DD_13_-RIP peptide MIC was the lowest, at 27.5 μM, and these results suggest that even at 13.8 μM, this peptide can greatly reduce the bioburden of the hydrogels. If total peptide concentration is a concern for any application, DD_13_-RIP-laden hydrogels may be an appropriate option.

At the MIC, indolicidin hydrogels had patches of dead cells, but many live bacterial cells remained, while at 0.5× MIC there was a minimal killing effect (Figure 3B). At 2× MIC, there was a drastic decrease in viability. Quantification of these findings showed a 55% and 88% drop in viability for indolicidin hydrogels formed with 1× and 2× soluble MICs, respectively, relative to AMP-free control hydrogels (Figure 3E). These results highlight the ability of indolicidin to reduce bacterial burden within these hydrogels. As a potential clinical application, there would be a requirement for a substantial reduction in bacterial viability, so it would be advisable to employ double the MIC concentration or higher. The 2× MIC corresponds to 88 μM of indolicidin, a concentration that is well-tolerated by human cells, as validated by a mammalian cell live–dead assay discussed below.

Starting at the MIC, the P10 AMP hydrogel demonstrated a pronounced decrease in the number of viable cells (Figure 3C). The normalized viability in P10-functionalized hydrogels was reduced from highly viable in 0.5× MIC hydrogels to just 1.6% viable in 1× MIC hydrogels, and this bactericidal effect was retained for P10 hydrogels formed at 2× MIC (Figure 3F). Taken together, our findings demonstrate that these three AMPs, when tethered to a hydrogel surface, retain excellent antimicrobial activity, and can prevent MRSA biofilms at or below their individual MICs. In addition, our results demonstrate that in vitro MIC screening with soluble AMPs can be used to create effective, antimicrobial AMP-loaded hydrogels. These findings have relevance in healthcare settings, where these AMP-laden hydrogels can be used as implant coatings that prevent biofilm-based infections of strains specific to the hospital where the device is being implanted.

### 2.4. Combinatorial AMP Hydrogels Are Effective against MRSA

**Figure 4:**
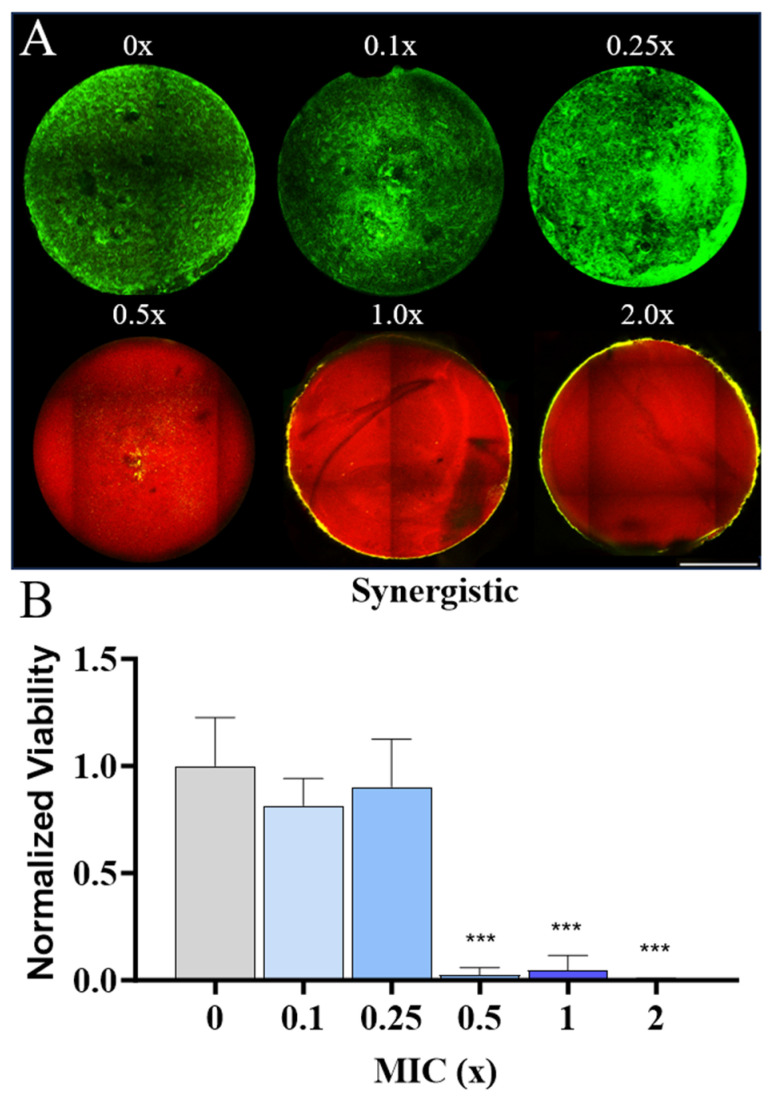
Synergistic effects of P10 and indolicidin on viability of MRSA atop different AMP-loaded hydrogels. (**A**) Qualitative analysis showing representative hydrogels functionalized with different fractions of the synergistic concentration for indolicidin and P10 (3.125 μM and 12.5 μM, respectively) and stained for live (**green**) and dead (**red**) cells. Scale bar: 1 mm. (**B**) Quantitative analysis of synergistic AMP hydrogel on biofilm viability. Significance (*** *p* < 0.001) in viability was established by comparing the test group to a control hydrogel group with no AMP (0×).

The use of AMPs at doses which display a synergistic effect has the potential to reduce the risk of antimicrobial resistance development, and by tethering these peptides to hydrogels the dosage can be held consistent in the targeted area without the need to overcompensate for clearance [64]. Towards this end, we explored coupling multiple AMPs to norbornene-modified hyaluronic acid (HANor) hydrogels at concentrations that yielded a synergistic effect against MRSA based on our checkerboard-array screening data. From these experiments, the combination of 3.125 μM indolicidin and 12.5 μM P10 yielded a synergistic effect against MRSA and an additive effect against MSSA. We tested multiples of these concentrations, which represented a proportional change of 0.1×, 0.25×, 0.5×, 1× and 2× synergistic concentrations (Figure 4). At low P10 and indolicidin concentrations representing 0.1× and 0.25× synergistic concentrations, the multi-AMP hydrogels formed had no effect on MRSA viability (Figure 4A). At P10 and indolicidin AMP concentrations above 0.5× synergistic concentrations, hydrogels completely killed the bacteria (Figure 4A). This can be seen visually in Figure 4A, which shows the switch from entirely green (live) to entirely red (dead) starting at the 0.5× MIC, and quantitatively in Figure 4B, which shows a statistically significant decrease in viability compared to the AMP-free control group. This observed effect at 0.5× MIC is reflective of a concentration of only 1.56 μM and 6.25 μM for indolicidin and P10, respectively. We observed that the MIC of indolicidin was 43.8 μM and for P10 it was 120 μM when used alone against MRSA (Table 1). When used in combination, they were effective at ~28- and ~20-fold-lower concentrations, respectively. As seen here, the combination of AMPs allows for the use of the antimicrobial agent at lower effective concentrations, which could reduce potential mammalian cell cytotoxicity associated with AMPs at high concentrations. In addition, using two different AMPs with different mechanisms of action reduces the risk of developing AMP resistance. These findings highlight the value of our high-throughput assay in identifying AMP combinations that minimize the total concentration needed to kill a specific pathogen and prevent biofilm formation for use in AMP-loaded hydrogels.

### 2.5. Single AMP-Loaded Hydrogels at the MIC and Combinatorial AMP Hydrogels Reduce Bacterial Bioburden on and within Hydrogels

Inspired by the findings that single-AMP and dual-AMP hydrogels at their soluble MIC exhibited excellent antimicrobial activity, we were interested in evaluating bacteria–AMP hydrogel interactions on a finer scale. Towards this end, hydrogels without AMPs or functionalized with DD_13_RIP, indolicidin, P10, or synergy (P10 and indolicidin) at their soluble MIC were formed. MRSA were cultured atop the hydrogels as described previously; two days later, the samples were stained for Syto-9 (green, live) and propidium iodine (red, dead), and high-resolution z-stacks (60× magnification, surface-imaged for every 1 µm of the hydrogels for 5 µm) were acquired to evaluate 3D viability and extent of bacteria infiltration into the hydrogels.

**Figure 5:**
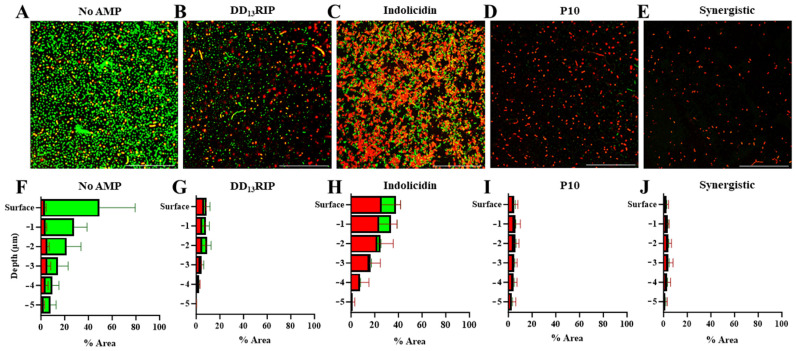
Investigation of MRSA cells interacting with AMP-hydrogels. Representative high-resolution (60× magnification) images of hydrogels tethered with (**A**) no AMP, (**B**) DD_13_RIP, (**C**) indolicidin, and (**D**) P10 at their soluble MIC, and (**E**) indolicidin and P10 at their synergistic soluble MIC. Scale bars: 100 µm. Quantitative spatial viability analysis of MRSA as a function of hydrogel depth of hydrogels tethered with (**F**) no AMP, (**G**) DD_13_RIP, (**H**) indolicidin, and (**I**) P10 at their soluble MIC, and (**J**) indolicidin and P10 at their synergistic soluble MIC.

On AMP-free hydrogels, live bacteria covered about 46% of the hydrogel surface (Figure 5A,F). In contrast, AMP-functionalized hydrogels all showed fewer live cells at the hydrogel surface. DD_13_RIP-functionalized hydrogels (27.5 µM) showed a lower total bacterial load on the surface compared to the no-AMP group with 7% covered by dead cells and 2% covered with live bacteria (Figure 5B,G). However, the total surface coverage of bacteria on indolicidin-functionalized hydrogels (43.8 µM) was highest among other AMPs (37%), and the majority of these cells (70% of surface bacteria) were dead (Figure 5C,H). P10-functionalized hydrogels (120 µM) were best among single-AMP hydrogels at preventing initial bacterial adhesion at the surface, and less than 1% of the cells bound to the hydrogel surface had living MRSA (Figure 5D,I). P10 prevents bacterial biofilm formation by disrupting the bacterial cell membrane [27], which results in the near complete elimination of bacteria on the hydrogel surface at the MIC. The synergistic hydrogel (3.1 µM indolicidin, 12.5 µM P10) showed essentially no live bacterial cells at the surface, and just 2% of the surface contained cells, almost all of which were dead (Figure 5E,J). These findings suggest that these dual-AMP hydrogels leverage the bactericidal effects of indolicidin-functionalized hydrogels and the anti-biofilm effects of P10-functionalized hydrogels, allowing for maximal antibacterial properties at a significantly lower total peptide concentration.

At a 5 μm depth inside AMP-free hydrogels, about 6% of the area was covered with living MRSA cells. While it is unlikely that the bacteria can immediately infiltrate the hydrogel, Gram-positive bacteria such as MRSA can secrete hyaluronidases and degrade hyaluronic acid, allowing them to burrow into hyaluronic acid hydrogels over time [65]. None of the AMP-loaded hydrogels showed a greater than 1% area of live cells within the gel at 5 μm depth. The AMP-loaded hydrogels appear to prevent the process of bacteria making their way below the hydrogel surface either by killing the bacteria on the surface or preventing them from collecting on the surface at all. Overall, the antimicrobial properties of all these peptides appear to function for improving the natural antimicrobial properties of hyaluronic acid and minimize the bacterial burden at the surface and within the hydrogel.

### 2.6. Mammalian Cells Are Viable on Single and Combinatorial AMP Hydrogels

**Figure 6:**
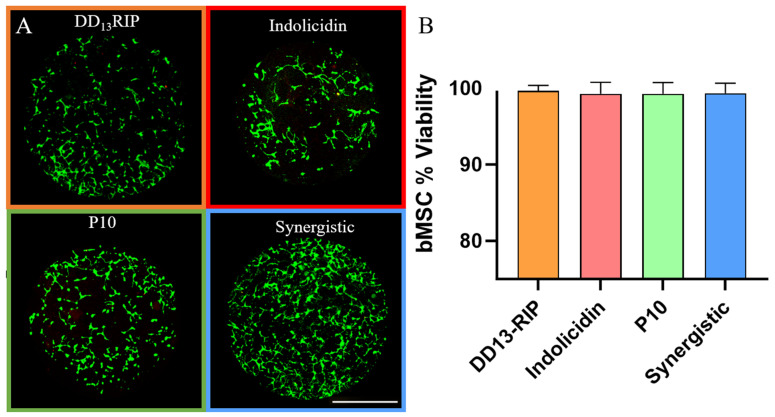
Mammalian MSC viability on AMP-loaded hydrogels. (**A**) Representative live–dead stain of MSCs seeded on AMP hydrogels at 2× MIC for each group. Scale bar: 1 mm. (**B**) Quantification of the viability of mammalian cells seeded on top of each AMP hydrogel. There were no significant differences in viability observed.

For establishing whether these AMP-loaded hydrogels could be used in practical biomedical applications, we looked at the effect that they had on mammalian cell viability. Mesenchymal stem cells (MSCs) were seeded onto RGD-functionalized (2 mM, to allow for MSC adhesion) AMP hydrogels at the highest concentration, 2× MIC for each AMP, as well as the synergistic concentrations for P10 and indolicidin. All AMP hydrogels promoted MSC cell attachment, growth, and spreading at these concentrations, as evidenced by the coverage and spread of live (green) MSCs on each hydrogel group (Figure 6A). For every 100 cells counted across each AMP group there was, at most, one cell which displayed a nuclear red stain, resulting in viability at or above 99% (Figure 6B). These data demonstrate that even at double the concentration required for antimicrobial properties, the tethered AMPs do not display significant cytotoxic effects. Thus, using our high-throughput screening methodology, AMP-loaded hydrogels could be created for infection prevention, providing an effective alternative to antibiotics, minimizing the potential for resistance development, while allowing for the growth of healthy mammalian cells. An additional benefit of utilizing low concentrations of AMPs is that the norbornene molecules are not saturated within these hydrogels, which would allow for the conjugation of additional thiolated molecules alongside these AMPs. For example, the addition of anti-inflammatory peptides could be useful for wound repair applications while preventing infection.

## 3. Materials and Methods

### 3.1. Materials

Resin (ProTide Rink) and all amino acids used for peptide synthesis were purchased from CEM Corporation (Matthews, NC, USA). Sodium hyaluronate (molecular weight: 66,000–99,000 Da) was purchased from Lifecore Biomedical (Chaska, MN, USA). Dowex Resin (50WX8, 100–200 mesh) was obtained from Thomas Scientific (Swedesboro, NJ, USA). 5-norbornene-2-methylamine, Spectra/Por dialysis tubing (6–8 kDa molecular weight cutoff), and tetrabutylammonium hydroxide (0.4 M) were obtained from VWR International (Radnor, PA, USA). FilmTracer, LIVE/DEAD™ Biofilm Viability Kit was obtained from Thermo Fisher (Waltham, MA, USA). Human bone-marrow-derived mesenchymal stem cells were obtained from Lonza Bioscience (Walkersville, MD, USA). All other chemicals and solvents were aquired from Sigma Aldrich (St. Louis, MO, USA) and used as received.

### 3.2. Peptide Synthesis

The peptides DD_13_-RIP (sequence: ALWKTLLKKVLKAYSPWTNF), indolicidin (sequence: ILPWKWPWWPWRR), and P10 (sequence: LAREYKKIVEKLKRWLRQVLRTLR) were prepared using a solid-state peptide synthesizer (Liberty Blue, CEM). The sequence GCGGG was added to the N-terminus of these peptides to provide a thiol group (in cysteine, C) for covalent binding to norbornene groups in the hydrogel network, and a flexible linker (GGG) to prevent steric hindrance between the thiol-norbornene bond and the functional domain of the peptide. Peptides were cleaved from the resin (ProTide Rink, CEM) using a solution of 92.5% trifluoroacetic acid, 2.5% triisopropylsilane, and 2.5% 2,2-(Ethylenedioxy)diethanethiol for 3 h. The peptides were precipitated three times in cold diethyl ether and lyophilized to improve storage and sterility. Peptide synthesis was confirmed using MALDI-TOF (matrix-assisted laser desorption/ionization time-of-flight) spectroscopy (Figure S4).

### 3.3. Bacterial Strains, Media, and Growth Conditions

De-identified patient isolates of MSSA and MRSA strains were obtained from Cooper University Hospital. These strains were acquired during routine diagnostic workup in the Clinical Microbiology Laboratory and the strains were identified using a VITEK (bioMerieux) system, MALDI-TOF mass spectrometry, and susceptibility testing. As no private identifiable patient information or human interaction was required, sample collection was exempt from IRB review. Bacterial strains were grown overnight at 37 °C, with aeration in lysogeny broth (LB) or Mueller–Hinton broth (MHB), when appropriate. The optical density at 600 nm (OD_600_) was determined and cells were diluted to a starting OD_600_ to 0.05 in MHB for MIC determination and checkerboard assays, and to 0.1 in Trypticase soy broth (TSB) for biofilm growth. For biofilm growth, 1 mL of the diluted bacterial culture was added to each well of a 24-well plate containing a hydrogel mounted on a glass coverslip and incubated for 2 days at 37 °C, and samples were then stained and prepared for confocal microscopy.

### 3.4. Determination of the Minimum Inhibitory Concentration (MIC)

MIC determination was performed by broth microdilution according to standard protocols [56,61,66]. Briefly, each soluble AMP was added to the first column of a 96-well plate and serially diluted 2-fold, ten times. The starting concentrations for the AMPs were 100 μM for DD_13_-RIP and 50 μM for indolicidin and P10. These values were selected using previously determined MICs as a guide [27,33,36], where the starting value was at least 16-fold higher, to allow for differences to be observed. Following dilutions, 50 μL of diluted MSSA or MRSA cells were added. Plates were incubated overnight at 37 °C, without shaking. The following morning, the OD_600_ was read in a Synergy plate reader (Biotek, Winooski, VT, USA). The MIC was determined as the lowest concentration of AMP where no bacterial growth was observed (OD_600_ < 0.1).

### 3.5. Checkerboard Assays

**Figure 7:**
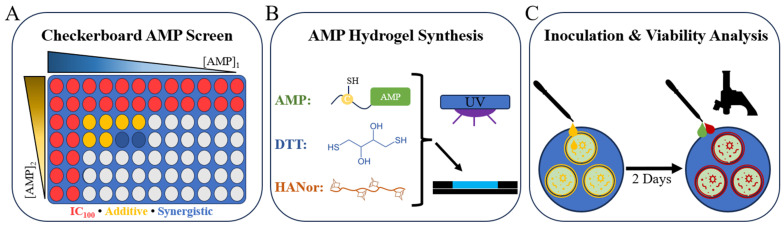
Schematic of the experimental design. (**A**) Diagram of 96-well plate array used for high-throughput screening of AMPs at different concentrations to determine additive or synergistic concentrations against MRSA and MSSA. Representative inhibitory (IC_100_), additive, and synergistic combinations are displayed. (**B**) Chemical components used to synthesize AMP hydrogels. (**C**) MRSA inoculation, incubation, staining, and imaging process diagram.

Checkerboard assays were carried out as described previously [61]. Briefly, each pairwise combination of AMPs was tested against MSSA and MRSA strains. The first AMP was added to the first column of the 96-well plate and diluted horizontally, as described for MIC determination. The second AMP was added to the first row and serially diluted 2-fold vertically (Figure 7A). A total of 50 µL of cells was added to the plate and analyzed, as described above. To determine potential additive or synergistic effects, the fractional inhibitory concentration index (FICI) was calculated using Equation (1):

(1) $$FICI=\frac{MIC\;A_{A+B}}{MIC\;A}+\frac{MIC\;B_{A+B}}{MIC\;B}$$

In this formula, MIC A_A+B_ and MIC B_A+B_ are the MICs of the AMPs in combination and MIC A and MIC B are the MICs of each AMP alone. If the FICI was above 4.0, the AMPs at those concentrations were antagonistic. If the FICI was between 1 and 4, the AMPs in combination had no effect. Additive effects are defined by the combination of AMPs producing a larger effect than either alone and are present when the FICI is between 0.5 and 1. Synergistic effects, as defined by the combination of AMPs producing a significantly larger effect than either alone, are denoted by FICI values less than 0.5.

### 3.6. HANor Macromer Synthesis

HANor was synthesized as described previously [51]. Briefly, sodium hyaluronate (NaHA, 2% *w*/*v*, molecular weight: 66–99 kDa) was mixed with Dowex resin in distilled water for two hours at room temperature. The resin was then vacuum filtered, and tetrabutylammonium hydroxide (TBA-OH, ~2.4 mmol per gram of NaHA) added to form HA tetrabutylammonium salt (HA-TBA). The resulting HA-TBA solution was then frozen and lyophilized. Carboxyl groups in the HA-TBA were then modified with norbornenes via amidation with 5-norbornene-2-methylamine in an anhydrous solution of dimethyl sulfoxide (DMSO, 2% *w*/*v*) and benzotriazole-1-yl-oxy-tris-(dimethylamino)-phosphonium hexafluorophosphate (BOP) under nitrogen for two hours at room temperature. The reaction was quenched with ice-cold water, dialyzed (SpectraPor, 6–8 kDa molecular weight cutoff), frozen, and lyophilized to improve storage and sterility. The synthesized HANor macromer had ~45% of its repeat units functionalized with norbornene, as confirmed with ^1^H NMR spectroscopy (Figure S5).

### 3.7. AMP Hydrogel Synthesis

Using aseptic techniques in a laminar flow hood, HANor macromers (5 wt%) were mixed in filter-sterilized phosphate-buffered saline (PBS) with di-thiol crosslinkers (dithiothreitol, DTT at 8 mM concentration), photoinitiator (Irgacure 2959, 0.05 wt%), appropriate thiolated AMP concentrations, and injected into UV-sterilized cylindrical molds (3 mm diameter, 0.8 mm height) (Figure 7B). The hydrogels were photopolymerized by irradiating with ultraviolet light (10 min, 10 mW cm^−2^) and washed twice with sterile PBS. Compression testing showed that the stiffness of the formed hydrogels ranges from 27.9 to 36.2 kPa, with P10 and DD_13_-RIP hydrogels being slightly stiffer than the other peptide-loaded hydrogel groups (Figure S6). Using soluble AMP screening data, hydrogels containing DD_13_-RIP, indolicidin, or P10 at 0.5×, 1.0×, or 2.0× their MIC against MRSA (9 groups) were synthesized. A synergistic combination consisting of indolicidin and P10 was identified against MRSA and hydrogels were formed with both AMPs at 0.1×, 0.25×, 0.5×, 1×, and 2× synergistic concentrations (Figure 7C). AMP-free hydrogels were also formed as peptide-free controls. For MSC viability studies, hydrogels were formed with 2 mM thiolated RGD peptide for adhesion and DD_13_-RIP, indolicidin, or P10 at double their MIC, or a synergistic two-AMP combination at double their synergistic concentrations to evaluate AMP effects on mammalian cell adhesion and viability.

### 3.8. Biofilm Viability

Biofilms were washed three times with sterile water to remove unattached cells. The live/dead biofilm viability assay (FilmTracer, Invitrogen) was performed according to the manufacturer’s instructions. A total of 200 µL of staining solution was added to each well and plates were incubated for 20 min in the dark at room temperature. Following incubation, wells were washed once with 1 mL of deionized sterile water to remove excess staining solution. The hydrogel assembly was then placed upside down on a thin glass coverslip and imaged using an inverted confocal microscope (Nikon A1R) within two hours of staining. For each hydrogel, macroscopic images were collected where a 5 × 5 mm^2^ tile scan image with a z-depth of 250 µm (step size of 10 µm) was acquired, and the green and red channel-acquisition parameters were kept constant throughout all experiments. The images were analyzed using ImageJ software (version 1.54h) by extracting 100 × 100 μm^2^ square image stacks from each hydrogel image stack (n = 4 per hydrogel), identifying focal plane image(s) within the z-stack, and by applying an Otsu threshold to create binary images from the green (live) and red (dead) channels. Twelve cross sections taken from the three hydrogels of each sample group were analyzed in this manner. Viability (% viable) was then quantified using Equation (2):

(2) $$\%\;viable=\left(1-\frac{Red\;\%\;Area}{Green\;\%\;Area}\right)\times 100$$

The % viable values were then normalized to the average viability from the control samples with no peptide, such that 100% represents a typical bacterial death rate on these hydrogels over the course of the experiment, as shown in Equation (3):

(3) $$Normalized\;viablity=\frac{Sample\;\%\;viable}{Control\;\%\;viable}\times 100$$

Additional hydrogels at 1× the MIC and synergistic concentrations were generated in the same manner and imaged at 60× magnification to visualize individual bacteria. Z-stacks (step size 1 µm) were taken of the surface and 5 µm into the hydrogel. An Otsu threshold was used to create binary images from the green (live) and red (dead) channels to calculate the % area covered by bacteria at each focal plane.

### 3.9. Mammalian Cell Viability

Human bone-marrow-derived mesenchymal stem cells (MSCs, Lonza, passage 4) were seeded on RGD-functionalized (2 mM) AMP hydrogels at a density of 20,000 cells cm^−2^. MSCs were incubated at 37 °C, 5% CO_2_ for two days in growth medium (α-minimum essential medium supplemented with 10% FBS (fetal bovine serum) and 1% penicillin/streptomycin). Three hydrogels were seeded for each AMP group. After two days, a Mammalian cell LIVE/DEAD Viability/Cytotoxicity Kit (Invitrogen) was constituted in growth medium, as per the manufacturer’s instructions. Samples were exposed to the staining solution for 30 min, and then the constituted live/dead solution was replaced with growth medium, and samples were imaged within 2 h. Live and dead cells were quantified by counting green and red individual cells, respectively. Live cells were quantified by hand by selecting four 1 × 1 mm^2^ image sections for each hydrogel group (n = 12 images analyzed per group). Viability was calculated by dividing the number of living cells by the total cell count.

### 3.10. Statistical Analysis

All experiments were carried out at least three independent times. Data were analyzed by one-way analysis of variance (ANOVA) and tested versus control hydrogels using Dunnett’s multiple comparisons test. Bar graphs represent the mean, and the error bars represent ± standard deviation. Differences among groups are stated as * *p* < 0.05, ** *p* < 0.01, and *** *p* < 0.001.

## 4. Conclusions

In this study, checkerboard arrays were used to identify soluble concentrations of DD_13_-RIP, indolicidin, and P10, which yielded combinatorial effects against MSSA and MRSA strains. The soluble screens identified a synergistic combination of P10 and indolicidin at a concentration 10- and 14-times lower than their MICs, respectively. These soluble-AMP screening data were then used to design hydrogels with individual AMPs at their MIC, and we found that these hydrogels successfully prevented MRSA biofilm formation. While these AMP concentrations are already significantly lower than many other AMP-hydrogel platforms, we also found that AMP hydrogels with indolicidin and P10 at half of their synergistic concentration were also highly effective in preventing MRSA adhesion and biofilm formation. By using soluble-screening AMP data, we rationally designed hydrogels with AMP dosing tailored to the pathogen of interest. Mammalian cells also adhere to, and are viable on, these AMP-laden hydrogels, demonstrating that the AMPs selectively thwart bacterial colonization while maintaining high biocompatibility. It is important to note that this checkerboard-based AMP screening technique can be applied to additional bacterial strains beyond MSSA and MRSA, to discover novel AMP combinations that selectively target pathogens of interest. In this study, AMPs were covalently bound to norbornene-modified hyaluronic acid hydrogels, and the thiol–norbornene chemistry used can be applied to functionalize any norbornene-containing material with thiolated AMPs. This new paradigm for AMP-hydrogel design has potential for designing biocompatible, antimicrobial materials with applications such as wound dressings and medical-device coatings, preventing biofilm formation of opportunistic pathogens.

## Figures and Tables

**Table 1:** Average minimum inhibitory concentration of each AMP against *S. aureus* strains.

AMP	MSSA *	MRSA *
DD_13_-RIP	13.5 μM	27.5 μM
Indolicidin	18.8 μM	43.8 μM
P10	87.5 μM	120 μM

* MIC determinations were made at least three independent times.

## Data Availability

The original contributions presented in the study are included in the article/Supplementary Material, further inquiries can be directed to the corresponding authors.

## Supplementary Materials

The following supporting information can be downloaded at: https://www.mdpi.com/article/10.3390/pharmaceutics16070860/s1, Figure S1: Optical density readings used to calculate combinatorial effects of soluble AMPs against MSSA; Figure S2: Optical density readings used to calculate combinatorial effects of soluble AMPs against MSSA; Figure S3: HANor hydrogel incubated for 2 days in PBS absent of bacteria and stained using the live/dead biofilm viability assay per the manufacturer’s instructions; Figure S4: Thiolated AMP MALDI mass spectrometry results; Figure S5: 1H NMR spectra of HANor macromer; Figure S6: Elastic modulus of AMP hydrogels.
