# Supplementary material for: Antimicrobial Peptide Screening for Designing Custom Bactericidal Hydrogels
Source: Pharmaceutics. 2024 Jun 27;16(7):860. doi: 10.3390/pharmaceutics16070860 (PMC11279943; doi:10.3390/pharmaceutics16070860)
Supplement: Supplementary file 1 [file pharmaceutics-16-00860-s001.zip › pharmaceutics-3066353-supplementary.pdf]

Article

Antimicrobial Peptide Screening for Designing Custom Bactericidal Hydrogels

Matthias Recktenwald [1], Muskanjot Kaur [2], Mohammed M. Benmassaoud [1], Aryanna Copling [3], Tulika Khanna [4], Michael Curry [2], Dennise Cortes [2], Gilbert Fleischer [2], Valerie J. Carabetta [2,*] and Sebastián L. Vega [1,5,*]

[1] Department of Biomedical Engineering, Rowan University, Glassboro, NJ 08028, USA
[2] Department of Biomedical Sciences, Cooper Medical School of Rowan University, Camden, NJ 08103, USA
[3] Department of Translational Biomedical Sciences, Rowan University, Glassboro, NJ 08028, USA
[4] Department of Biological Sciences, Rowan University, Glassboro, NJ 08028, USA
[5] Department of Orthopedic Surgery, Cooper Medical School of Rowan University, Camden, NJ 08103, USA
* Correspondence: carabetta@rowan.edu (V.J.C.); vegas@rowan.edu (S.L.V.)

MSSA

A

Concentration Indolicidin (µM) \ Concentration P10 (µM)	100	50	25	12.5	6.25	3.125	1.56	0.78	0.39	0
25	0.051	0.058	0.060	0.052	0.063	0.055	0.060	0.062	0.057	0.058
12.5	0.052	0.085	0.058	0.049	0.048	0.050	0.050	0.046	0.045	0.043
6.25	0.058	0.070	0.053	0.046	0.044	0.044	0.043	0.043	0.043	0.042
3.13	0.053	0.069	0.071	0.046	0.044	0.042	0.041	0.096	0.170	0.288
1.56	0.072	0.070	0.057	0.049	0.043	0.043	0.042	0.457	0.423	0.400
0.78	0.810	0.070	0.054	0.047	0.044	0.401	0.420	0.406	0.450	0.443
0.39	0.081	0.068	0.571	0.491	0.438	0.447	0.402	0.551	0.510	0.430
0	0.068	0.072	0.350	0.766	0.630	0.567	0.601	0.684	0.779	0.625

B

Concentration DD_{13}-RIP (µM) \ Concentration P10 (µM)	100	50	25	12.5	6.25	3.125	1.56	0.78	0.39	0
50	0.092	0.075	0.059	0.060	0.071	0.079	0.083	0.077	0.084	0.520
25	0.099	0.062	0.054	0.051	0.048	0.050	0.052	0.049	0.059	0.059
12.5	0.099	0.058	0.057	0.048	0.048	0.044	0.043	0.044	0.059	0.046
6.25	0.110	0.074	0.060	0.047	0.047	0.045	0.048	0.044	0.495	0.498
3.125	0.095	0.395	0.471	0.514	0.574	0.568	0.505	0.488	0.565	0.494
1.56	0.091	0.506	0.629	0.665	0.513	0.497	0.549	0.463	0.437	0.497
0.78	0.110	0.637	0.507	0.658	0.546	0.572	0.536	0.577	0.531	0.508
0	0.124	0.796	1.230	0.967	0.786	0.764	0.789	0.839	0.825	0.875

C

Concentration Indolicidin (µM) \ Concentration DD_{13}-RIP (µM)	50	25	12.5	6.25	3.125	1.56	0.78	0.39	0.20	0
25	0.086	0.093	0.064	0.062	0.066	0.057	0.059	0.100	0.060	0.062
12.5	0.082	0.082	0.061	0.073	0.417	0.305	0.470	0.542	0.396	0.469
6.25	0.070	0.066	0.057	0.056	0.467	0.561	0.501	0.550	0.700	0.704
3.125	0.056	0.068	0.059	0.054	0.545	0.523	0.554	0.575	0.618	0.589
1.56	0.056	0.067	0.056	0.051	0.647	0.572	0.626	0.598	0.545	0.593
0.78	0.083	0.069	0.056	0.097	0.662	0.505	0.563	0.564	0.666	0.545
0.39	0.080	0.061	0.057	0.052	0.591	0.622	0.455	0.526	0.641	0.587
0	0.051	0.069	0.058	0.124	1.062	0.945	1.042	1.157	1.030	1.078

Inhibitory
Synergistic
Additive
Growth

Fig. S1. Optical density readings used to calculate combinatorial effects of soluble AMPs against MSSA (Fig. 2). The MIC for each individual peptide is highlighted in grey and corresponds to the lowest concentration which results in no growth (bold box). The red colored boxes indicate wells with no bacterial growth ($OD_{600} < 0.1$) and green colored boxes indicate bacterial growth. The box in the bottom right corner contains no drug and serves as a growth control, yellow shaded boxes indicate additive interactions and blue boxes indicate synergistic interactions based on FICI calculations.

MRSA

A

Concentration P10 (µM) \ Concentration Indolicidin (µM)	25	12.5	6.25	3.125	1.56	0.78	0.39	0.20	0.098	0
100	0.052	0.086	0.077	0.065	0.092	0.068	0.080	0.067	0.067	0.077
50	0.054	0.088	0.065	0.056	0.053	0.048	0.051	0.090	0.055	0.048
25	0.050	0.081	0.067	0.057	0.048	0.065	0.325	0.327	0.355	0.467
12.5	0.052	0.087	0.079	0.061	0.201	0.435	0.380	0.466	0.410	0.378
6.25	0.047	0.184	0.147	0.518	0.537	0.487	0.505	0.623	0.760	0.719
3.125	0.066	0.225	0.558	0.626	0.613	0.583	0.460	0.707	0.838	0.720
1.563	0.045	0.305	0.587	0.647	0.574	0.586	0.694	0.853	0.825	0.608
0	0.045	0.403	0.864	1.271	1.150	1.072	1.018	1.043	0.991	0.961

B

Concentration P10 (µM) \ Concentration DD_{13}-RIP (µM)	100	50	25	12.5	6.25	3.125	1.56	0.78	0.39	0
100	0.123	0.080	0.066	0.064	0.070	0.060	0.058	0.072	0.064	0.058
50	0.137	0.065	0.048	0.054	0.051	0.051	0.054	0.056	0.051	0.063
25	0.116	0.065	0.066	0.048	0.045	0.047	0.046	0.064	0.258	0.286
12.5	0.122	0.065	0.073	0.047	0.045	0.044	0.324	0.371	0.507	0.495
6.25	0.131	0.075	0.062	0.048	0.049	0.167	0.458	0.464	0.584	0.561
3.13	0.122	0.077	0.051	0.051	0.054	0.374	0.384	0.524	0.527	0.661
1.56	0.114	0.062	0.069	0.058	0.071	0.408	0.365	0.516	0.640	0.635
0	0.134	0.056	0.054	0.056	0.222	0.699	0.688	0.771	0.761	0.683

C

Concentration Indolicidin (µM) \ Concentration DD_{13}-RIP (µM)	50	25	12.5	6.25	3.125	1.56	0.78	0.39	0.20	0
25	0.059	0.088	0.087	0.063	0.071	0.064	0.064	0.063	0.064	0.063
12.5	0.047	0.082	0.061	0.054	0.049	0.046	0.046	0.078	0.145	0.146
6.25	0.078	0.086	0.052	0.047	0.045	0.044	0.272	0.380	0.290	0.346
3.125	0.080	0.084	0.050	0.045	0.043	0.213	0.425	0.356	0.347	0.391
1.56	0.076	0.076	0.051	0.044	0.045	0.323	0.449	0.462	0.472	0.449
0.78	0.055	0.054	0.055	0.048	0.049	0.370	0.571	0.461	0.452	0.390
0.39	0.071	0.052	0.054	0.054	0.456	0.585	0.522	0.540	0.539	0.661
0	0.066	0.069	0.067	0.066	0.473	0.815	0.660	0.580	0.830	0.898

Inhibitory
Synergistic
Additive
Growth

Fig. S2. Optical density readings used to calculate combinatorial effects of soluble AMPs against MRSA (Fig. 3). The MIC for each individual peptide is highlighted in grey and corresponds to the lowest concentration which results in no growth (bold box). The red colored boxes indicate wells with no bacterial growth (OD_{600} < 0.1) and green colored boxes indicate bacterial growth. The box in the bottom right corner contains no drug and serves as a growth control, yellow shaded boxes indicate additive interactions and blue boxes indicate synergistic interactions based on FICI calculations.

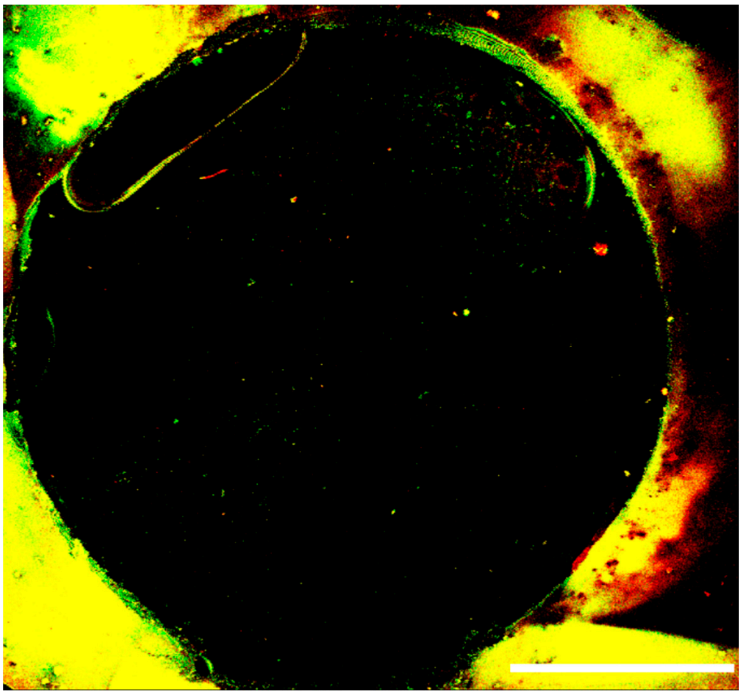

Fig. S3. HANor hydrogel incubated for 2 days in PBS absent of bacteria and stained using the live/dead biofilm viability assay per the manufacturer's instructions. No green or red dye was visible within the circular gel area during imaging indicating that the stains are interacting with the bacteria and not the gel itself. Scale bar: 1 mm.

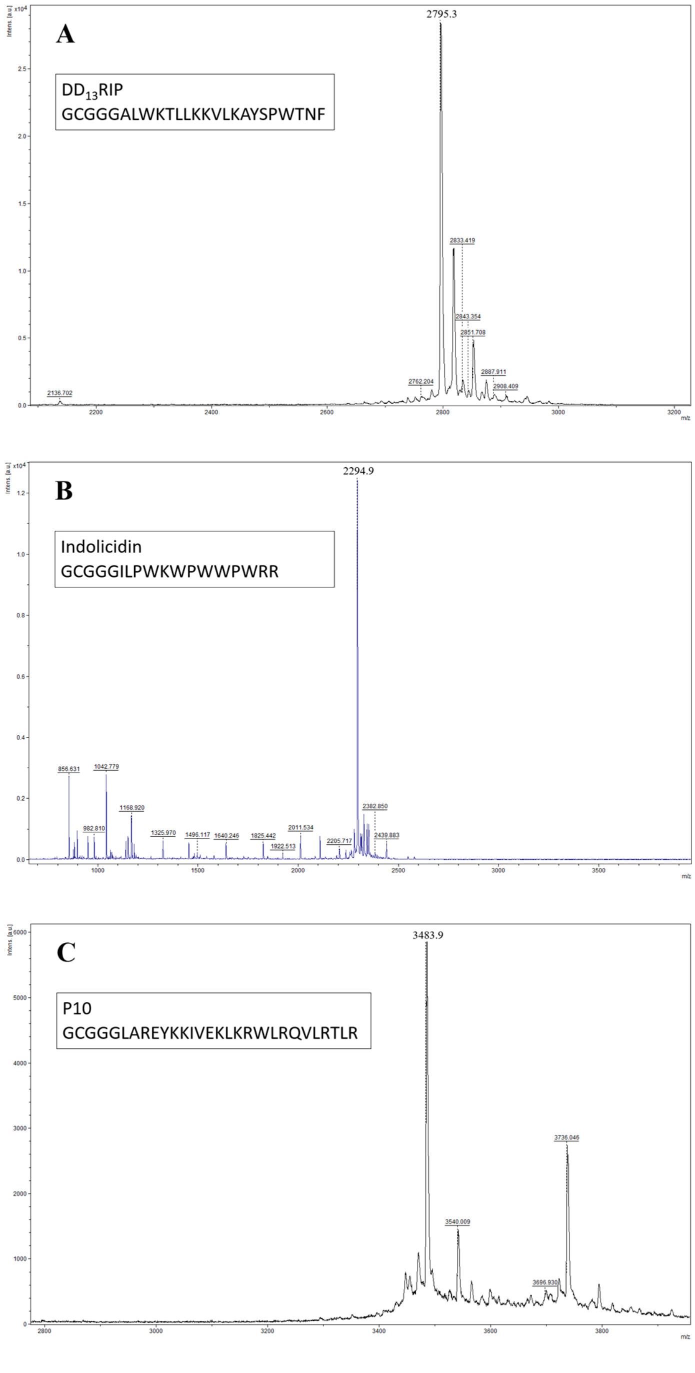

Fig. S4. (A) Thiolated DD13-RIP MALDI mass spectrometry results. Most intense peak: 2795.3 *m/z*; expected peak: 2794.3 *m/z*. (B) Thiolated indolicidin MALDI mass spectrometry results. Most intense peak: 2294.9 *m/z*; expected peak: 2295.0 *m/z*. (C) Thiolated P10 MALDI mass spectrometry results. Most intense peak: 3483.9 *m/z*; expected peak: 3484 *m/z*.

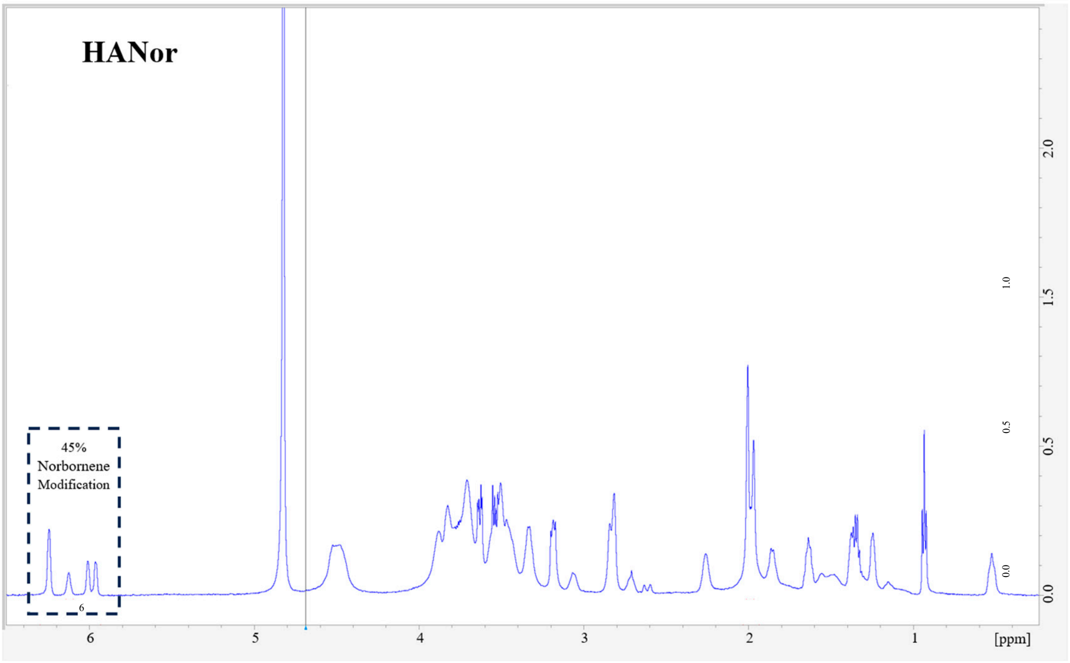

Fig. S5. ^{1}H NMR spectra of HANor macromer.

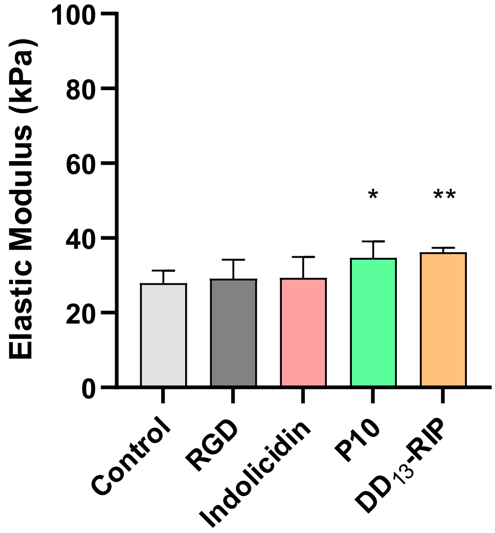

Fig. S6. Elastic modulus of AMP hydrogels. Hydrogels were formed by creating hydrogel solutions (5 wt% HANor, 8 mM DTT, and 2 mM RGD or the AMPs at 2x MIC) which were placed in cylindrical molds (8 mm diameter, 2.0 mm height), and photopolymerized with UV light (10 mW/cm^2, 10 minutes). Formed

hydrogels (n=6 per group) were then taken out of the molds and allowed to swell overnight in PBS. All groups were compared using an ANOVA test, with each test group compared directly to the control group, and the Dunnett correction for multiple comparisons was applied. The stiffness was significantly higher for the P10 (* $p = 0.0357$) and DD_{13}-RIP (** $p = 0.0084$) groups. This increase in stiffness corresponds to peptides with the longest amino acid chains and highest molecular weights. There are likely intramolecular forces or protein entanglements causing this slight increase in hydrogel stiffness.
